# Take me on a ride: The role of environmentalist identity for carpooling

**DOI:** 10.1002/mar.21340

**Published:** 2020-02-18

**Authors:** Barbara Hartl, Bernadette Kamleitner, Sandra Holub

**Affiliations:** ^1^ Department of Marketing Vienna University of Economics and Business Vienna Vienna Austria; ^2^ Department for Management and Economics Danube University Krems Krems Lower Austria Austria

**Keywords:** carpooling, environmentalist, identity, peer‐to‐peer platform, sharing economy

## Abstract

Sharing does not need to involve corporate providers but can also happen on a peer‐to‐peer (P2P) basis. P2P sharing platforms who match private providers and users are thus dealing with two different customer segments. An example of this is carpooling, the sharing of a car journey. Recent years have seen considerable research on why people use sharing services. In contrast, there is little knowledge of why people may offer a good for sharing purposes. Drawing on identity theory, this paper suggests that users and providers of carpooling need to be addressed differently. A pilot study and two studies, including both actual car owners and nonowners confirm that the extent to which one identifies as an environmentalist predicts car owners' willingness to offer carpooling, but does not affect nonowners' willingness to use carpooling services. These findings remain robust when controlling for various potential confounds. Furthermore, Study 2 suggests that an environmentalist identity plays an important role for car owners' actual decision to offer a ride via an online platform. These results suggest that marketers of P2P platforms need to pursue different strategies when addressing potential users and providers on the same platform.

## 1 INTRODUCTION

The term “sharing economy” refers to a multitude of different consumption practices (Belk, 2014) covering a range of services in almost all domains of consumption, predominantly mobility and accommodation (e.g., Abrahao, Parigi, Gupta, & Cook, 2017; Bridges & Vasquez, 2018; Cannon & Summers, 2014; Gunter, 2018; Lee, Chan, Balaji, & Chong, 2018; Midgett, Bendickson, Muldoon, & Solomon, 2018; Ndubisi, Ehret, & Wirtz, 2016; Yang, Lee, Lee, & Koo, 2018; Zervas, Proserpio, & Byers, 2014). The sharing economy can enable the use of underutilized goods without invoking the transfer of individual ownership and thus, can add to efficient resource use (Heinrichs, 2013). Especially in the transportation industry, various new businesses have recently disrupted traditional modes of travel. While some of these new sharing businesses might have resulted from a need for cost reduction after the global economic crises in the late 2000s, their success was also accelerated by a growing awareness of environmental problems combined with the ubiquity of information and communication technologies (Cohen & Kietzmann, 2014). These technologies enable sharing on a large scale and allow companies to reach new market segments (Casprini, Paraboschi, & Di Minin, 2015). Sharing platforms are organizing the sharing process and help consumers to gain short‐term access to shared transportation modes. BlaBlaCar, for instance, is a peer‐to‐peer (P2P) platform organizing carpooling, that is, the sharing of a trip so that more than one person travels in a car (Casprini et al., 2015). The role of the platform is to match supply and demand; thus, private individuals can offer empty seats in their car for a trip on the platform and the platform connects them with riders.

The fact that carpooling platforms do not own the shared vehicles distinguishes them from other forms of shared mobility organized as business‐to‐consumer services, where the shared cars and bicycles are usually owned by the operating platforms. This results in certain challenges for the marketing of carpooling. P2P carpooling platforms serve two segments of users, drivers who provide a ride in their car and riders who use the offered ride (cf. Hartl, Penz, Schüßler, & Hofmann, 2019). Previous research in the sharing economy has focused on users of sharing services. Their motives to use sharing services of products they do not own are well investigated (Hamari, Sjoklint, & Ukkonen, 2016; Hartl, Sabitzer, Hofmann, & Penz, 2018; Hawlitschek, Teubner, & Gimpel, 2016). A common finding is that economic and pragmatic rather than environmental and idealistic considerations dominate the decision to consume sharing services. Carpooling platforms, however, not only need to target users of rides, but they also need to target car owners offering those rides. Essentially, the car owners' provision determines the quality and quantity of the service offered. Yet, to date, there is little insight into what makes owners offer rides via a carpooling platform. Existing insights into the motivations of those who do not own cars, may not generalize to those that do own cars. A comprehensive perspective on carpooling that acknowledges the totality of potential customers is needed to identify ways of successfully targeting both users and providers. Building on research on identity‐based consumer behavior (Reed, Forehand, Puntoni & Warlop, 2012), on notions of extended self (Belk, 1988), and the social identity theory (Tajfel, 1974), this paper aims to contribute to such a perspective.

The main premise of the current research is that the decision on whether or not to provide the owned good for sharing purposes turns into a decision of identity‐relevance. As possessions are a major contributor to and reflection of our identities (Belk, 1988), providing an owned good for sharing purposes entails providing something that, via ownership, has been connected to the self and contributes to identity. In contrast, deciding on whether or not to temporarily use a good is of lesser relevance to the user's identity. In other words, users and providers may differ with regard to the identity‐relevance of their respective sharing behavior. Notably, consumers' self‐concept contains multiple identities and is in particular determined by what social groups and purposes a person identifies with. Given that sharing has often been framed as a sustainable and environmentally friendly practice (Heinrichs, 2013), the current research specifically focuses on the extent to which consumers' identify as an environmentalist. Based on previous literature it is suggested that the degree to which people endorse an environmentalist identity influences car owners' decision to provide carpooling but it does not influence nonowners' decisions to demand carpooling rides. A pretest and two online surveys with actual car owners and nonowners support this prediction. Notably, the evidence is still found even if other known predictors of sharing behavior and variations in the usage of the target good, that is, car usage preferences and habits, are controlled.

In summary, this paper contributes to existing literature and informs managerial practice in the following ways. First, this paper extends the existing body of knowledge by introducing considerations of identity as a relevant factor of owners' intention to provide a good for sharing purposes. Second, by situating this study in the context of carpooling, this paper provides much‐needed insights into this specific and under‐researched domain. New forms of shared mobility such as carpooling via P2P platforms have emerged over the last years and have enabled shared mobility between strangers. Due to this change in how carpooling is organized through a two‐sided platform, research on consumers' engagement is needed to provide insights for P2P platform marketing. Third, as a result of focusing on a particular aspect of identity, this paper shows that an environmentalist identity drives sharing behavior. Notably, this provider‐based insight on the role of sustainability contrasts with prior insights on user motivations. Thus, the current research contributes to the ongoing discussion on the role of environmental concerns as motives to engage in sharing behavior. Finally, this paper deduces concrete and novel implications to tackle the marketing challenges faced by P2P platforms. An empirical analysis of the impact of identity can provide valuable insights as to why people are willing to offer P2P sharing services. It thereby advances the understanding of drivers in the sharing economy in general and of carpooling in particular. It also provides relevant insights into whether marketing efforts aiming at increasing participation rates in carpooling can be generalized across the target groups of providers and users or whether different message strategies need to be developed for these groups.

### 1.1 Conceptual and theoretical background

In the sharing economy, digital platforms, such as BlaBlaCar, organize sharing and allow for interaction between individuals from all over the world. This offers new possibilities for the practice of sharing, which originally was limited to a small group of people living close to each other (Belk, 2014). Platforms provide infrastructure and rules, acting as an intermediary between the private individuals providing a good or service and the private individuals consuming the shared good or service. Importantly, P2P sharing platforms serve two key types of customers: providers and users. They thereby form what has been called a two‐sided platform by Eisenmann, Parker, and Van Alstyne (2006). In two‐sided platforms, more demand from one user segment spurs more demand from the other user segment (Eisenmann et al., 2006). For instance, the more car owners register at a P2P platform and the more rides they offer, the more users register at the platform because the enhanced offers more likely fit their needs (e.g., concerning departure point and destination). In turn, the more users are registered at a platform, the more private car owners may be willing to offer a ride because it is more likely that they find a suitable passenger. Value thereby grows as the platform matches demand from both sides (Eisenmann et al., 2006). Two‐sided platforms are not a new phenomenon; however, due to the ongoing technological developments, such P2P platforms have become more prevalent and now allow for more substantial resource savings.

Mobility, together with accommodation, is the most promising area in the sharing economy (Cohen & Kietzmann, 2014). Urban mobility is a major challenge for cities, due to fast population growth and environmental problems (Katzev, 2003). Shared mobility services seem an auspicious tool to help address these problems as currently the majority of private vehicles are not used to their full capacity. The majority of trips are single‐occupant vehicle trips, which result in a higher number of cars for the same number of riders (Correia & Viegas, 2011). The average occupancy rate of private cars is rather low (about 1.6 people per vehicle; Andersen, Lundli, Holden, & Høyer, 2004), yet the uses are high. This constitutes a resource‐saving potential that could be exploited through higher adoption rates of P2P carpooling. In the following section, a review of the literature on carpooling, an overview of identity theory and a review of influencing factors of sharing is provided.

#### 1.1.1 Carpooling

Carpooling is a particularly resource‐efficient mode of sharing and different from carsharing. In carpooling as opposed to carsharing, consumers do not share access to cars; instead, they share a ride in the same car. On carpooling platforms, private car owners usually post their journeys and offer empty seats in their cars for a planned trip. Users, who would like to travel somewhere, can then decide to book an empty seat offered on the platform. Thus, P2P carpooling platforms also need to be distinguished from household‐based, or internal carpools (Vanoutrive et al., 2012). Carpooling via P2P platforms takes time for picking up a passenger, whereas members of the same household have the same departure point in common. Carpooling platforms, such as BlaBlaCar, operate worldwide but do not own cars themselves. Rather, they make use of this underutilized resource and act as a mediator between private individuals pooling their needs. BlaBlaCar, as the leading European P2P carpooling platform, was valued at $1.5 billion in 2015 (Schechner, 2015). Carpooling platforms, such as BlaBlaCar or Zego, forbid for‐profit drivers. This distinguishes carpooling from profit‐oriented ride hiring (cf. Uber; Moore & Barbour, 2016). In the principles of carpooling, resource efficiency is center stage. Drivers have to declare that the price of the ride only covers their costs like fuel and maintenance. Monetary considerations of cost‐sharing can still play a role for providers but they are likely of lesser importance compared to ride hiring or taxi services (cf. Ravenelle, 2017). Overall, carpooling is considered a sustainable form of transportation (Caulfield, 2009) and sustainability may be recognized as an asset of carpooling.

Previous research on carpooling is scarce and has focused in particular on company‐led carpooling initiatives in a work context. Because employees of the same company share the same way almost daily, appeals made through companies are particularly effective in encouraging carpooling (Horowitz & Sheth, 1977; Kurth & Hood, 1977). Many of the scientific studies on carpooling were conducted towards the end of the previous century. A key insight was that the most common objection of solo drivers to carpooling is a lack of flexibility (e.g., Giuliano, Levine, & Teal, 1990; Glazer, Koval, & Gerard, 1986; Kearney & De Young, 1995; Teal, 1987). However, this research was conducted before the emergence of the internet and P2P platforms. Much has changed since then. One recent contribution to carpooling by Bachmann, Hanimann, Artho, and Jonas (2018) acknowledges the role of online carpooling platforms by particularly addressing the role of norms for offering a ride as a driver and accepting a ride as a passenger via an online platform. Online platforms have introduced new levels of flexibility via the sheer amount of opportunities offered and they have made it much easier to share a ride. In addition, the public concern for environmental problems increased over the last decades, moving environmental issues from a fringe to a mainstream issue (Kalafatis, Pollard, East, & Tsogas, 1999), including in the domain of transportation.

Despite all these developments, the fact remains that P2P carpooling forbids for‐profit drivers and that it may somewhat compromise car owners' flexibility (e.g., they need to stick to a predetermined schedule if they take on a rider). A question that previous research cannot yet sufficiently answer is how, despite these hurdles, private individuals can be attracted to offer their private cars and rides to P2P carpooling platforms. Thus, the current research focuses on the impact of consumers' identity on the willingness to provide carpooling, in particular, consumers' identity as an environmentalist.

#### 1.1.2 The role of identity in sharing

Following the definition by Reed et al. (2012, p. 312), identity can be defined as “any category label with which a consumer self‐associates.” In line with research on identity theory (Reed et al., 2012; Tajfel & Turner, 1985), people classify themselves into categories, yearning to acquire and maintain a positive identity. What consumers do is related to their identity and expression of identities; their decisions of buying, using, or disposing products and services is driven by their identities. Their identity thus affects their perception, emotions and behavior (Ellemers, Spears, & Doosje, 2002), and who one is and how people view themselves influences what actions they take (Aaker & Akutsu, 2009). Marketing practitioners have long known that consumers' sense of who they are influences their consuming decisions (Reed & Bolton, 2005). The decision to buy certain products and also the decision to not buy them express who they are. It is thus likely that peoples' view of themselves, that is, their self‐experienced identity, plays a crucial role in whether or not they will volunteer sharing a good that to some degree is part of their self. In that sense, an identity approach (Hogg & Reid, 2006; Reed et al., 2012) may provide a suitable analytical angle for understanding private individuals' intention to share their goods.

Notably, the notion of identity consists of multiple facets and is in particular determined by what social groups and purposes a person identifies with. People categorize themselves on the basis of demographics, social roles, or shared consumption patterns or preferences (Reed & Bolton, 2005). Some of these identities are relatively stable, while others may be subject to transitory, for instance, consumers' identity as an “athlete identity” or”mac‐user” (Reed et al., 2012). As previous research has discussed the role of environmental motives for participating in the sharing economy, the current research focuses on the consumers' identity as an environmentalist, thus, the extent to which they identify as an environmentally friendly person. A literature overview on the role of sustainable motives for participating in the sharing economy reveals contradicting results (see Table 1).

**Table 1:** Literature overview on sustainability in the sharing economy

Source	Sharing practice	Business model	Perspective	Findings on sustainability
Ballus‐Armet, Shaheen, Clonts, and Weinzimmer (2014)	Carsharing	P2P	Provider/user	Apart from monetary reasons and other benefits, environmental benefits were raised by potential vehicle renters and potential vehicle provider as positive perceptions of P2P carsharing.
Hamari, Sjöklint, and Ukkonen (2015)	General	P2P	User	Sustainability is not directly associated with participation in collaborative consumption, only when associated with positive attitudes towards collaborative consumption
Hartl et al. (2018)	Carsharing	B2C/P2P	User	Sustainability is rather perceived as an indirect consequence of carsharing
Hawlitschek et al. (2016)	Rental services	P2P	Provider/user	Sustainability is identified as a potential motive for using and providing peer rental services, but it is not included in the tested model as it mixes with modern lifestyle and thriftiness
Heinrichs (2013)	General	P2P/B2C	/	Sharing economy is discussed as having the potential to provide a new pathway to sustainability
Hellwig, Morhart, Girardin, and Hauser (2015)	General	P2P	Provider/user	Four potential clusters of sharing consumers suggested: sharing idealists, sharing opponents, sharing pragmatists, and sharing normatives. Sharing businesses suiting the nature of the “sharing normative” might be those businesses that feature large social and/or environmental benefits that can be reaped in a publicly visible way.
Martin (2016)	Online sharing economy	P2P	/	An analysis of the online sharing economy discourse leads to the identification of six different frames. The sharing economy is framed as (a) an economic opportunity; (b) a more sustainable form of consumption; (c) a pathway to a decentralised, equitable and sustainable economy; (d) creating unregulated marketplaces; (e) reinforcing the neoliberal paradigm; and, (f) an incoherent field of innovation.
Möhlmann (2015)	Carsharing and accommodation	P2P/B2C	User	Environmental impact has no effect on the satisfaction and the likelihood of choosing a sharing option again.
Piscicelli, Cooper, and Fisher (2015)	General/borrow each other's objects, spaces and skills	P2P	Provider/user	“To be green” is stated as main reason for joining a specific P2P marketplace (Ecomodo) by one‐third of respondents. Values, such as benevolence and universalism represent the sample respondents' main priorities, with universalism‐concern and universalism‐tolerance ranking better than universalism‐nature.
Tussyadiah and Pesonen (2018)	Accommodation sector	P2P	User	Sustainability as part of social appeal is discussed as driver for using P2P accommodations.
Wilhelms, Henkel, and Falk (2017)	Carsharing	P2P	Provider	Most important drivers for providing carsharing: economic interests (“earn”), quality of life (“enjoy”), helping others (“enrich”), and sustainability (“enhance”); sustainability is seen as an indirect consequence

Abbreviation: P2P, peer‐to‐peer.

Table 1 shows that though environmental motives were rarely observed to actually trigger sharing behavior, in particular by users of shared services, there is no doubt that sharing is often considered as environmentally friendly. Moreover, research on the role of sustainability has been focusing on different sharing practices, such as sharing accommodations or carsharing (Table 1). It has to be taken into account that carsharing, that is, temporarily giving away one's car for users' sole usage, differs from carpooling, that is, giving someone a lift in one's car, insofar as sharing a car comprises less sustainable potential than a joint trip to the same location. This is where the current research sets in. To engage in sharing a ride via carpooling could, therefore, help to express a person's identity as an environmentally friendly person (i.e., environmentalist identity). The identity as an environmentalist can be described as part of an individual's self‐concept. It is the extent to which people see themselves as the type of person who acts environmentally friendly (cf. Prati, Albanesi, & Pietrantoni, 2017). Environmentally friendly identities such as “environmentalist” (Lacasse, 2013) or “green identity” (Whitmarsh & O'Neill, 2010) have been identified as a key predictor of political engagement and activism or proenvironmental behavior such as carbon offsetting behavior. This should hold particularly if identity becomes a salient context. As outlined, this is likely for providers who share an identity‐related good. The extent to which they subscribe to an environmentalist identity is likely to influence their willingness to share identity‐relevant goods. This should hold in particular in carpooling where the provided good tends to be of identity‐relevance, where the sharing actually amounts to an act of sustainable resource‐saving and where overriding economic motives are likely absent.

The fact that previous research on P2P sharing suggests that environmental concerns play a minor role compared to economic considerations may be owed to the domination of the users' perspective of sharing in sharing economy research. For many activities in the sharing economy, users can save a relatively large amount of money by using a shared service instead of buying the good. If at all, environmental reasons may be particularly important for providers of shared goods. For private providers, the economic gains for sharing their goods or offering a service are often small in comparison to the purchase price of the good, which may result in a difference in motivations between providers and users of goods (cf. economic motivations, Böcker & Meelen, 2017). Based on these arguments, the present research proposes the following hypotheses:

**H1**: *The intention to provide carpooling services (but not the intention to use carpooling services) is influenced by an environmentalist identity*.
**H2**: *Car owners' decision to offer a ride via a P2P carpooling platform is influenced by an environmentalist identity*.

#### 1.1.3 Influencing factors of sharing goods

As it is possible that the identity as environmentalist relates to several other factors that may also drive the sharing behavior of car owners, controlling for other influencing factors is important. The broader literature on the sharing economy highlights the diversity of factors that influence sharing behaviors. Although consumers' environmental concerns may play a role, other factors have to be taken into account when focusing on carpooling and will be considered as control variables in the analysis. Indeed, it is important to consider that the current identity‐based account should be robust to demographic variations and reflect them only insofar as specific identities may be differentially important for different groups of people. For instance, where one lives tends to influence the willingness to share or pool a car with others (e.g, Neoh, Chipulu, & Marshall, 2017) and the distance to work may affect possible rides of a certain length. Further, Neoh et al. (2017) showed in their meta‐analysis that women are in general more likely to carpool than men, although in the past it was suggested that women are less likely to form nonhousehold carpools than men due perhaps to household commitments that do not correspond to the inflexibility of carpooling (Ferguson, 1995). The current research builds on the prediction that the effect of an environmentalist identity holds even after controlling for such demographic factors.

##### Car usage

In addition to demographic variables, the characteristics of the shared object matters. With carpooling, the object of sharing is one of great importance. A car is a particularly meaningful possession; cars have been used for purposes of social categorization and for esteem‐enhancement (Sowden & Grimmer, 2009). Building on identity theory, consumers hold multiple identities and related to their car ownership may identify as “motorists.” Past research has shown that consumers draw on identities such as “motorist” and “pedestrian” in describing their reaction to travel planning initiatives (Gardner & Abraham, 2007; Murtagh, Gatersleben, & Uzzell, 2012), which may be of importance for carpooling. Some car drivers will identify stronger as motorists than others. In line with the identity theory, a strong motorist identity affects consumers' decisions and behavior. For instance, when consumers identify as “motorists,” travel demand management policies restricting car traffic are likely to evoke negative responses (Gardner & Abraham, 2007). While in the current research environmentalist identity is suggested to be more relevant for the intention to provide carpooling, the actual driving may make the identity as motorist salient. Notably, the identity as environmentalist and identity as a motorist might result in an identity conflict (Reed et al., 2012). Thus, the current study sets into consideration to what extent carpooling intention and behavior are influenced by identity as an environmentalist when controlling for the identity as a motorist. To do so, the identity as a motorist is assessed, that is, the extent to which a person identifies as a car driver (cf. Murtagh et al., 2012).

To allow for encompassing controls several other different potential confounds related to car usage behavior are addressed. First, it is possible that people with strong environmentalist identity bond less with their car and thus are more willing to share it because they care about it less. To address this possibility the analysis controls for psychological car ownership (Kamleitner & Feuchtl, 2015). The notion of psychological ownership refers to individuals' feelings that a target product is theirs (Pierce, Kostova, & Dirks, 2001). There is growing evidence that consumers can develop strong possessive feelings towards products (Jussila, Tarkiainen, Sarstedt, & Hair, 2015). Research on carsharing has demonstrated that psychological ownership of products plays a significant role when consumer decide whether to share a product: Psychological ownership has been identified as moderator for the effects of instrumental car attributes on consumers' intention to select a shared car (Paundra, Rook, van Dalen, & Ketter, 2017). Thus, car owners' psychological ownership of their car is included in the current studies to assess whether the effect of identity on carpooling behavior still holds if controlled for car owners' attachment to their car. Second and related to the previous point, it is possible that people who are willing to provide their car to carpooling simply derive more pleasure from having a company during a ride or from driving a car in general. Consumers differ in whether they enjoy driving a car or not, which is for instance reflected in their transportation behavior (cf. van Exel, De Graaf, & Rietveld, 2011; von Behren et al., 2018). The current research builds on the prediction that the effect of an environmentalist identity holds even after controlling for variables related to car usage.

##### General attitudes

Consumers may provide carpooling due to a general attitude toward consumption, such as anticonsumption attitudes or general environmental concerns, rather than the enjoyment or pleasure derived from having company while driving. Consumers may provide carpooling because they simply want to enable others to drive somewhere without having to buy a car. In this sense, sharing has been related to anticonsumption, which can be described as “being against consumption” (Ozanne & Ballantine, 2010). Thus, noncar owners might refrain from buying a car, just like car owners might refrain from using a car alone due to anticonsumption considerations. To disentangle such general attitudes from the effect of identity, attitudes towards anticonsumption and general environmental concerns are assessed and controlled for (Iyer & Muncy, 2009). The current research builds on the prediction that the effect of an environmentalist identity holds even after controlling for general attitudes.

#### 1.1.4 Overview of studies

In the following, a pilot study and two survey studies are presented as part of two larger research projects on consumers' travel behavior and the sharing economy. A pilot study was conducted to test the impact of environmental identity taking into account sex, age, income, and distance to work (control variables: *demographic variables*). In addition to controlling for demographics, Study 1 controls for several potential confounds that ensures that any potential effect of an environmentalist identity does not reflect other potential factors (control variables: *demographic variables, car usage, general attitudes*). While the sample in Study 1 consists of car owners and noncar owners, measuring the intention to provide or use carpooling services, the sample in Study 2 consists also of carpoolers recruited via carpooling platforms and thus allow to additionally investigate whether consumers who offered a ride via a carpooling platform differ in terms of their environmentalist identity from car owners, who have never used a platform for carpooling (control variables: *demographic variables, car usage, general attitudes*). This approach allows for a robustness check and a further test of the proposed account.

## 2 PILOT STUDY

A pilot study was conducted to assess the role of car owners' environmentalist identity for the intention to provide carpooling in contrast to potential riders (i.e., noncar owners; *H1*). A total sample of 168 university students and graduates completed an online questionnaire (*M*_age_ = 26.20 years; standard deviation [*SD*]_age_ = 6.22; 64.3% female; *M*_income_ = 951.50 EUR; *SD*_income_ = 630.25). Most participants (89.2%) held a driving license and nearly half of the sample (44.6 percent) owned a car at the time they participated in the study. Two binary logistic regression analyses separated for car owners and noncar owners investigating the role of environmentalist identity for the intention to carpool were calculated. In addition to environmentalist identity, *demographic variables* were added as control variables. In line with predictions, the analysis revealed environmentalist identity as a significant predictor for car owners (*p* = .015). Based on the odds ratio, every additional scale point on the identity scale (100‐point slider) meant that the likelihood of people indicating their intention to carpool raised by 3.5. Repeating the same analyses with noncar owners, that is, potential users of sharing in the form of carpooling, a different pattern of results are found. Notably, the full model for noncar owners was not significant and environmentalist identity had no significant effect on carpooling intentions (*p* = .120). Results of the pilot study support the prediction that the degree to which car owners subscribe to an identity as an environmentalist significantly predicts their willingness to provide their car for carpooling and environmentalist identity does not significantly impact the intention to use carpooling for noncar owners (*H1*; Table 2).

**Table 2:** Summary of logistic regression analyses: the effects of an environmentalist identity and of the control variables

	Pretest: intention to use/provide carpooling				Study 1: intention to use/provide carpooling				Study 2: intention to use/provide carpooling				Study 2: offering a ride via a platform	
	Noncar owners (potential users)		Car owners (potential providers)		Noncar owners (potential users)		Car owners (potential providers)		Noncar owners (potential users)		Car owners (potential providers)		Car owners	
	Odds ratio	*p*	Odds ratio	*p*	Odds ratio	*p*	Odds ratio	*p*	Odds ratio	*p*	Odds ratio	*p*	Odds ratio	*p*
Environmentalist identity	1.018	.166	1.035	.015	1.012	.270	1.021	.039	1.051	.447	1.188	.001	1.402	.077
Demographic variables														
Sex^a^	0.688	.550	2.010	.224	0.449	.124	0.386	.064	0.675	.065	0.553	.001	4.622	.012
Age	0.947	.405	0.952	.295	1.012	.537	0.994	.762	0.988	.101	0.980	.001	0.987	.507
Income	1.001	.241	1.000	.485	1.000	.876	1.000	.697	0.970	.635	1.041	.415	1.096	.532
Education^b^					2.685	.044	2.042	.188	1.505	.062	1.447	.030	0.751	.575
Distance to work/university	0.970	.251	0.970	.240	0.993	.757	1.023	.405	1.002	.614	1.000	.986	1.028	<.001
Car usage												
Motorist identity					0.995	.532	1.004	.700	1.040	.486	0.915	.033	0.929	.551
Enjoy driving with others					1.884	.024	1.119	.591	1.396	.004	1.193	.051	1.659	.102
Enjoy driving a car							1.001	.997			1.067	.498	0.891	.672
Psychological car ownership							0.770	.147			0.938	.192	0.939	.658
General attitudes														
Anticonsumption scale					0.790	.265	1.095	.685	1.453	.009	1.037	.721	0.797	.459
General environmental concern					1.352	.184	1.854	.028	0.709	.016	0.972	.772	1.465	.198

^a^ (0 = Male, 1 = female).

^b^ (0 = No high school diploma or university degree; 1 = high school diploma or university degree).

## 3 STUDY 1

### 3.1 Sample, design, and procedure

The sample consists of a representative population panel, which was recruited by a professional market research agency. To ensure that the sample consisted of potential providers and users, the sample contained quotas to ascertain an equal distribution along with this criterion but to keep demographics otherwise equal. An attention check was embedded in the beginning of the Study to reduce error variance and increase statistical power (cf. Oppenheimer, Meyvis, & Davidenko, 2009). The survey link was opened by 564 visitors of the Web site, out of which five participants closed the survey immediately before or after the first question. After the attention check (“*To test your attention, we ask you to do not mark the right answer for the question below, but to choose the sixth category Paris*”), 120 people were dismissed from the questionnaire. Out of 439 participants, 302 participants finished the questionnaire. One additional person was excluded due to conflicting answers (indicating “683” as age). No further exclusions were made. Thus, the final sample is composed of a diverse and representative sample of 301 Viennese adults (*M*_age_ = 41.17 years; *SD*_age_ 13.89, 51.8% female). The mean age of Vienna's population is 40.8 years (Statistik Austria, 2017), 51.3% of the Viennese population is female (Stadt, 2017). The mean income was 1.339,01 EUR (*SD* = 740.55). About half of the participants (46.2%) had children. Half of the participants (52.8%) were owning a car at the time they completed the Study. Table 3 depicts frequencies of participants' education and occupancy situation.

**Table 3:** Demographics of Studies 1 and 2

	Study 1		Study 2	
	Percentage	95% CI	Percentage	95% CI
Education				
Compulsory school	10.3	[7.2–14.1]	4.6	[3.5–5.9]
Apprenticeship degree	49.2	[48.2–59.4]	21.1	[18.8–23.6]
Higher school certificate	23.6	[19.1–28.6]	27.4	[24.8–30.0]
University degree	12.3	[8.9–16.4]	32.2	[29.5–34.9]
Other			14.8	[12.2–18.0]
Occupational situation				
School/study	11.0	[6.7–17.0]	9.7	[8.1–11.5]
Employed	49.8	[44.2–55.5]	58.7	[55.9–61.6]
Self‐employed/freelancer	5.9	[3.5–10.2]	11.5	[9.7–13.4]
Retired	16.6	[12.7–21.1]	15.6	[13.6–17.8]
Jobseeker	9.0	[6.1–12.6]	5.3	[4.1–6.7]
Other	7.6	[5.0–11.1]	5.7	[3.5–9.0]

Abbreviation: CI, confidence interval.

The survey contained questions regarding car ownership, identity, willingness to participate in carpooling, transportation behavior, psychological ownership of the car, anticonsumption and general environmental concern, as well as demographic variables (e.g., gender, income, children, and age). Car ownership was measured as a dichotomous variable (yes/no) and identity was measured on a 100‐point slider‐scale where the participants should rate how strongly they feel as an environmentally friendly person. Motorist identity was measured analogous to environmentalist identity on a 100‐point slider‐scale in which the participants should rate how strongly they feel as a motorist. Car owners' willingness to share their own car was measured after an introduction of the concepts of carpooling and carsharing with the question “Would you provide your car to others for carpooling, carsharing or both?” Both forms of sharing were described and assessed to ensure that participants would not confuse them. The binary variable “willingness to provide carpooling” was coded “yes” (when stating “for carpooling” and “for both”) and “no” (when stating “carsharing” or none) for further analysis. Noncar owners' willingness to use carpooling services was measured with the binary variable “Would you use carpooling services?” Further, transportation behavior was measured, in particular items assessing enjoyment of driving a car (“how much do you like driving a car?”, car owners only) and of driving with others (“how much do you like sharing a ride with someone else?”) were used. Both items were assessed on 5‐point smiley scales where the particularly sad smiley was coded as “1” and the particularly happy smiley was coded as “5”. There also were items assessing car owners' psychological ownership of their car, general environmental concerns, and anticonsumption attitudes. The additional variable psychological ownership was assessed using three items adapted from Kamleitner and Feuchtl (2015), e.g., “In my mind I feel the car is MINE.” (7‐point Likert Scale: 1 = “*totally disagree*,” 7 = “*totally agree*,” *α* = .714). Attitude for anticonsumption was measured using five items adapted from Iyer and Muncy (2009), for example, “If we all consume less, the world would be a better place” (7‐point Likert Scale: 1 = “*totally disagree*,” 7 = “*totally agree*,” *α* = .820). The assessment of general environmental concern was adapted from Zelezny, Chua, and Aldrich (2000; e.g., “The balance of nature is very delicate and easily upset”, 7‐point Likert Scale: 1 = “*totally disagree*,” 7 = “*totally agree*,” *α* = .798).

### 3.2 Results

To investigate the role of environmentalist identity for the intention to carpool (*H1*), two binary logistic regression analyses were again calculated separately for car owners and noncar owners (Table 2). The full model for car owners containing all predictors was statistically significant, *χ* ^2^ (*df* = 12) = 24.311, *p* = .018, (Nagelkerkes *R* ^2^ = .274). As predicted, and in support of Study 1, the analysis revealed environmentalist identity as a significant predictor. Based on the odds ratio, every additional scale point on the identity scale (100‐point slider) meant that the likelihood of people indicating their intention to carpool raised by 2.1. Further, general environmental concern predicted the willingness to provide carpool (see Table 1). Education, enjoyment of driving a car and driving with others, as well as the psychological ownership of the car, anticonsumption attitude and motorist identity are not statistically significant. However, a tendency effect of sex (*p* = .064) can be observed, indicating that men were more likely to provide carpooling than women.

As before, for noncar owners, less of an effect can be seen and the full model was not significant, *χ* ^2^ (*df* = 10) = 15.557, *p* = .113. Specifically, environmentalist identity had no significant impact on carpooling (see Table 2). However, the analysis revealed that enjoyment of driving with others is linked to the willingness to take offered ride shares: The more consumers enjoy to drive with others, the more they are willing to take a shared ride. Further, education (*p* = .044) significantly predicted the willingness to carpool.

Study 1 shows that an environmentalist identity impacts the willingness to provide carpooling, but not the willingness to use carpooling (*H1*). The effect of the identity as environmentalist still holds when controlling for variables related to car usage, such as enjoyment of driving a car, psychological ownership of the car, motorist identity, as well as general anticonsumption attitudes. Notably, it also holds when controlling for environmental concerns. This suggests that environmentalist identity as a driver of carpooling provision goes beyond any effects of proenvironmental attitudes.

## 4 STUDY 2

### 4.1 Sample, design, and procedure

A sample of 1,132 adults (*M*_age_ = 43.36 years; *SD*_age_ = 14.05, 49.6% female) was recruited via carpooling platforms from Austria and Germany and a research marketing agency and completed the questionnaire. About half of the participants stated to own a car (59.3%). Most of the participants reported an income between 1,501 and 2,000 EUR (20.6%). Table 3 depicts the frequencies of participants' education and occupancy situation.

**Figure 1:**
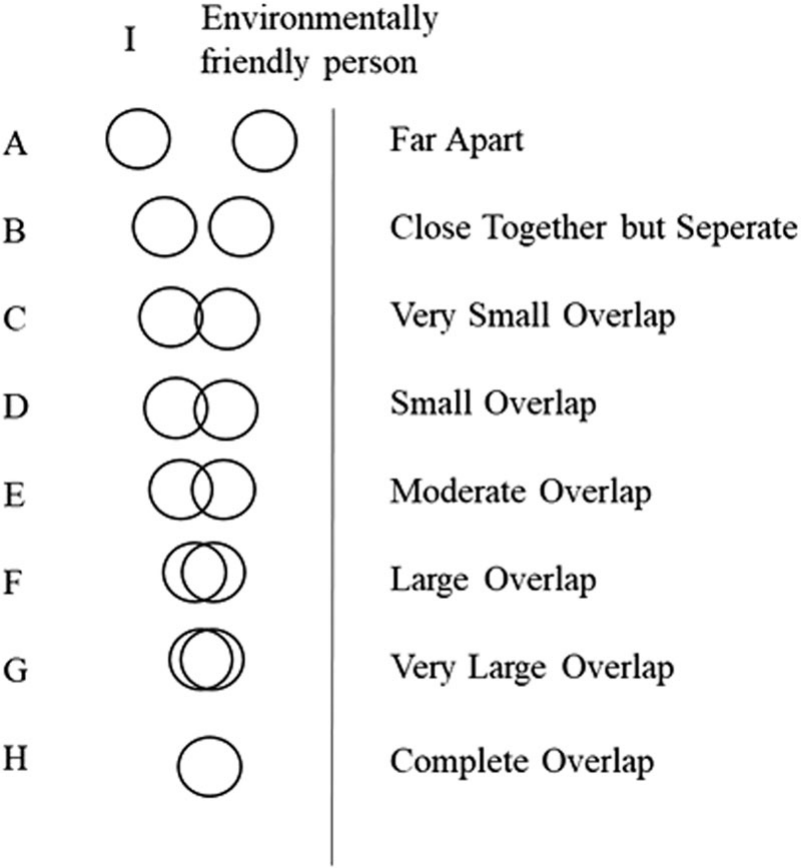
Measure of environmental identity used in Study 2 (adapted from Bergami & Bagozzi, 2000)

In Study 2, the same questions as in Study 1 were used to assess car ownership, transportation behavior, psychological ownership of the car, anticonsumption and general environmental concern, as well as demographic variables. The reliability of all scales was satisfying (*α* > .739) with the exception of the psychological ownership‐scale (*α* = .536), so that the item “I have the feeling that the car is something that someone can take from me” had to be removed (*α* = .838). The participants' intention to provide carpooling (“intention provider”) and their intend to use carpooling (“intention rider”), as well as their actual experience with carpooling (“Have you ever given someone a lift as part of carpooling?”) were measured as a dichotomous variable (yes/no). Further, for measuring identity a direct, graphically based measure was used adapted from Bergami and Bagozzi (2000; Figure 1).

### 4.2 Results

#### 4.2.1 Intention to provide or use carpooling in the future

To investigate the role of identity for the intention to carpool (*H1*), two binary logistic regression analyses were again calculated separately for car owners and noncar owners (Table 2). The full model for car owners containing all predictors was statistically significant, *χ* ^2^ (*df* = 12) = 52.772, *p* < .001, (Nagelkerkes *R* ^2^ = .104). As predicted, and in support of Study 1, the analysis revealed environmentalist identity as a significant predictor (*H1*). Based on the odds ratio, every additional scale point on the identity scale meant that the likelihood of people indicating their intention to carpool raised by 1.9. Further, motorist identity, education, sex, and age significantly predicted the willingness to provide carpool (see Table 2). Enjoyment of driving a car, enjoyment of driving with others, income, and distance to work, as well as the psychological ownership of the car, anticonsumption attitude, and general environmental concerns were not statistically significant. However, a tendency effect of enjoyment of driving with others (*p* = .051) can be observed.

The model for noncar owners' intention to use carpooling was significant, *χ* ^2^ (*df* = 10) = 32.135, *p* < .001. Environmentalist identity, as well as motorist identity, had no significant impact on carpooling (see Table 2). However, like in Study 1, the analysis revealed that enjoyment of driving with others is linked to the willingness to take offered ride shares: The more consumers enjoy driving with others, the more they are willing to take a shared ride. Further, anticonsumption attitudes and general environmental concerns significantly predicted the willingness to carpool and a tendency for sex and education was observed (Table 1). Thus, similar to Study 1, Study 2 shows that an environmentalist identity impacts the intention to provide carpooling, but not the willingness to use carpooling (*H1*) and that the effect of the identity as environmentalist holds when controlling for transportation behavior, such as enjoyment of driving a car, psychological ownership of the car, motorist identity, as well as general anticonsumption attitudes.

#### 4.2.2 Registration at carpooling platforms

To further identify the role of environmentalist identity for car owners' decision to offer a ride at carpooling platforms (*H2*), a binary logistic analysis was conducted. Participants, which were recruited via carpooling platforms where they were offering a ride, were compared to car owners, who stated they have never used a platform for carpooling purpose (Table 2).

The full model for car owners containing all predictors was statistically significant, *χ* ^2^ (*df* = 12) = 41.768, *p* < .001, (Nagelkerkes *R* ^2^ = .241). The analysis reveals a tendency effect of environmental identity (*p* = .077) after controlling for demographic variables, variables related to car usage, such as motorist identity, enjoy driving with others, enjoy driving a car, and psychological ownership, as well as attitudes towards anticonsumption and general environmental concerns. Thus, the analysis supports the previous findings that consumers' identification as environmentalist plays a role in their decisions to provide carpooling (*H2*). The analysis further shows that, among all variables, distance to work/university and sex play the most decisive and robust role. The domination of these two variables as the most significant variable continues as variables related to the sharing good and general attitudes were entered. If consumers have to commute a very long distance to work or university, they are more likely to offer a ride on a carpooling platform.

### 4.3 General discussion

Although P2P sharing platforms are receiving wide attention among practitioners and academia, research on factors driving private individuals' willingness to share their goods is remarkably rare. As the sharing of private goods has the potential to add to a more sustainable society (Heinrichs, 2013), consumers' green motives for engaging in the sharing economy have been debated over the last years. The purpose of the current research was to examine the role of identity for engaging in P2P carpooling. The results show that car owners' environmentalist identity is related to their willingness to contribute to the sharing economy by offering their car for carpooling purpose. This is consistent with prior research that finds that identity influences why people give (Aaker & Akutsu, 2009; Gerber, Hui, & Kuo, 2012), for instance, why people fund projects on crowdfunding platforms. Notably, the current research shows that findings generalize beyond a sample of those known as most likely to engage in the sharing economy to a population sample. Findings even remain robust if controlled for various potential confounds that may originate from people's riding habits, general attitudes towards mainstream consumption or their relationship to their car. The fact that environmentalist identity predicted the willingness to provide carpooling services even after controlling for environmental concerns further fosters the proposed role of identity.

## 5 THEORETICAL IMPLICATIONS

From a theoretical point of view, the current research contributes to identity theory (Reed et al., 2012) by examining the role of identity in the decision to share one's goods. People support efforts that are consistent with their identity; in the case of carpooling, sharing a ride is consistent with the identity as an environmentalist. Notably, results suggest that such consistency between intentions and identity still requires a trigger. The proposition made here is that the implication of an identity‐relevant good, here people's cars, acts as such a trigger. In line with this proposition, results show that the environmentalist identity of potential users that lack such a trigger is not related to their willingness to use carpooling services. Thus, the current results suggest that it is necessary to adopt a differentiated view on the role of environmental concerns in the sharing economy. While both users and providers are needed for P2P sharing to work, the decision to share ones' own possession appears to be driven by different factors than the decision to temporarily use products or services offered by others. This is similar to the way giving differs from taking (Clarke, 2006) and suggests that the impacting factors may differ. In light of these prior insights, the current research suggests that the distinguishing line between providers and users may largely reflect the relevance of the shared good to the self. Because many shared goods are meaningful possessions, their sharing is likely to increase considerations of the good but also of owners' own identity. As consumers' possessions represent their identities and are regarded as parts of themselves (Belk, 1988), consumers' decision to share a good becomes identity‐relevant, especially when the act of sharing is made public, such as providing carpooling by posting trips on P2P carpooling platforms. Thus, sharing of good increases considerations of the consumers' own identity. On the one hand, consumers express their identities by how and what they consume or not consume, and on the other hand, consumers constantly engage in a monitoring process in which they retrospectively inspect associations within their identity to make sure that they are behaving in a consistent manner (Reed et al., 2012).

As stated earlier, consumers' self‐concept contains multiple identities and thus, any identity is not possessed in isolation (cf. Reed et al., 2012). Related to the current research, a strong identity as environmentalist may result in an identity conflict if consumers at the same time identify strongly as a motorist, as driving a car is not perceived as sustainable and environmentally friendly. Research suggests that consumers seek to maintain harmony between their various identities and that consumption may not only lead to identity conflict, but provide ways to resolve it (Amiot, De la Sablonniere, Terry, & Smith, 2007; Reed et al., 2012). Carpooling may be a possibility to resolve the conflict between a strong environmentalist identity and a strong motorist identity: although in the current research, consumers' identity as motorist had no significant impact on the willingness to provide carpooling, consumers who identify strongly as an environmentalist and use their car to commute daily may provide carpooling to resolve a potential conflict.

Further, as a consumers' self‐concept contains multiple identities, different identities may be salient at different stages of the decision process or may be relevant for different decisions (cf. Reed et al., 2012). Thus, the impact of consumers' identity as an environmentalist may be different for information‐seeking on carpooling platforms, for registering on a carpooling platform, for the decision to actually offer a ride or for how often consumers actually offer a ride on a carpooling platform. This relates to the assumption of Hamari et al. (2015), arguing that although consumers might have started participating in the sharing economy for intrinsic reasons and perceived sustainability, the factors influencing their decision to continue participating might shift toward extrinsic ones.

## 6 PRACTICAL IMPLICATIONS

If policy makers endorse the support of carpooling programs, this study is holding strong implications. One of the most effective ways to exploit energy saving potentials would be for private companies or public institutions to implement carpooling programs or to make use of existing carpooling platforms. Private companies can offer commuting programs to work, whereas public institutions can focus on the connectivity of people in rural areas.

The question that arises is if there is a way to improve carpooling attractiveness for car owners and noncar owners by promoting this transport alternative. Unlike prior research, the current study examined this question from the perspective of the private provider. Based on the results of current research and the literature on carpooling, the following practical conclusions can be drawn for marketers:

### 6.1 Address carpoolers and riders differently

Carpooling P2P platforms represent two‐sided markets (Eisenmann et al., 2006; Rysman, 2009), thus, the platforms' marketing need to target two sets of agents, which interact through the platform: private car owners and users seeking a ride. The current study shows that what is relevant for the one customer segment may not be relevant for the other. Previous research has shown that while employees or founders of P2P services report to place great emphasis on idealistic motivations, such as sustainability, private users of the services, on the other hand, want to get what they need whilst increasing value and convenience (Bellotti et al., 2015). The results at hand are one of the rare contributions that suggest that idealistic motivations may actually extent to one target group of carpooling services, that is, providers, but not the other, that is, users. Thus, marketing campaigns need to consider both customer segments and feed them with different promotional messages, for example, having a welcoming homepage for both customer segments which makes it easy for both groups to maneuver to distinct areas oriented towards the target group. Rather than first showing some general information and then having those interested follow target group‐specific information, the landing page might be the best place to initiate that segmentation.

### 6.2 Target environmentalist identity of potential carpooler

Results at hand found little in the way of predicting why users would subscribe to carpooling. The one exception was the joy of riding with others which is clearly a message that carpooling platforms could leverage. Most insights were gained on car owners, that is, potential providers. Results show that providing one's car is also a matter of identity and in particular of environmentalist identity. The simplest step could be to simply stress that carpooling is, in fact, a more sustainable alternative to driving alone (Bachmann et al., 2018). Policy makers worldwide can use this aspect for systematic campaigning. Marketers could also target car owners' identity as an environmentalist, by displaying CO_2_ savings of their specific car when using carpooling in the platforms' app. Targeting the prototype of an environmentalist opens up a wide range of consumer and community‐based solutions for traffic problems.

## 7 FUTURE RESEARCH DIRECTIONS

The current research suggests that the minor role of environmental concerns in previous research may be owed to the domination of the users' perspective of sharing. Another possible reason is related to the ongoing professionalization of P2P sharing platforms and in particular the increase of the for‐profit component. This may result in a decrease of intrinsic motivation to provide sharing services analogous to the framing of the sharing economy as an economic opportunity (cf. Martin, 2016). In the early years of the sharing economy, carpooling websites were not managed professionally and the design was unappealing (cf. “looked like an Excel file,” Casprini, Di Minin, & Paraboschi, 2018). Nowadays, Blablacar is one of the world's largest carpooling platforms, leveraging on specific social media features and organizing demand and supply via a professional app for smartphones. Private users of sharing services may not even perceive relevant differences when booking a ride via Blablacar compared to calling a taxi using an app. The sharing economy market is especially experiencing professionalization of P2P sharing services which allow for‐profit usage. On Airbnb, private individuals offer their apartments next to professional rental service providers (Li, Moreno, & Zhang, 2015), sometimes taking out loans to buy apartments specifically to participate on the P2P sharing platform (cf. Ravenelle, 2017). Airbnb further offers a professional photo shooting for free, creating value for private providers (Airbnb, n.d.; Hein, Böhm, & Krcmar, 2018). From an identity perspective, this could mean that a special possession turns into an investment good. It would be of relevance for future research to test whether the addition of a for‐profit component undermines considerations of identity‐relevance. Although the current research underscores the importance of identity for P2P sharing, this may not be the case for platforms which allow or even encourage for‐profit usage. Ravenelle (2017) addresses this issue by using the term “sharing economy workers” to describe Uber drivers and Airbnb hosts and points out that whereas the main web presence of Uber focuses on convenience issues, the driver‐partner site is all about the income possibilities. Thus, the current research provides a starting point for examining changes in the consumers' motivation to offer and use sharing services analogous to the development and the professionalization of the P2P sharing practices. Although idealistic goals may be more prevalent at the outset of sharing initiatives, sharing platforms in a more professionalized stage may appeal to consumers with different motivations.

As with all research, some limitations need to be considered. The representative study was undertaken in a capital city in central Europe with a good public transportation system. Although P2P carpooling services seem to cover mostly trips from or to larger cities, carpooling platforms may contribute to solve the problem of aging of the rural populations by promoting new sales areas outside of cities. Age is thus another relevant consideration for future research. Population aging will give rise to a substantial increase in the numbers of older consumers, who's quality of life in advanced age is related to mobility (MacDonald & Hébert, 2010; Metz, 2000). Older people living in less densely populated areas may face difficulties making trips to the grocery store, medical treatments or social activities as they age further or develop medical conditions (Su & Bell, 2009). Another limitation concerns the measurement of the environmentalist identity: Although the term “environmentally friendly person” is a commonly used term, consumers may have slightly different understandings of the term. Future studies could take this into account by providing a definition before measuring the level of identification.

Finally, several research opportunities arise through the newly identified relevance of identity. This research focused on environmentalist identities but sharing plays into more than one identity. It would, for example, be relevant to see whether the identity as someone who helps or an efficiency‐seeker would show similar effects to that of environmentalist identity.
